# Borophene as a promising anode material for sodium-ion batteries with high capacity and high rate capability using DFT

**DOI:** 10.1039/c8ra01942h

**Published:** 2018-05-15

**Authors:** Jianhua Liu, Cheng Zhang, Lei Xu, Shaohua Ju

**Affiliations:** State Key Laboratory of Complex Nonferrous Metal Resources Clean Utilization, Kunming University of Science and Technology Kunming 650093 China; Faculty of Metallurgical and Energy Engineering, Kunming University of Science and Technology Kunming 650093 China kmustleixu@126.com xulei_kmust@aliyun.com kmustshaohuaju@126.com

## Abstract

Two-dimensional boron synthesized by the chemical vapor deposition method is an atomically thin layer of boron with both light weight and metallicity. To investigate the potential of borophene as an anode material in sodium-ion batteries, first-principles calculations and *ab initio* molecular dynamics simulations were carried out. The calculated results reveal that after introducing vacancy defects, the special puckered structure becomes relatively flat and the metallic nature of the defective borophene is enhanced, while the defects in borophene can weaken sodium adsorption. A single sodium atom is preferentially absorbed on the B_V_ site. The adsorption energies gradually reduce with an increase in sodium concentration due to the increased Na–Na repulsion. The fully sodium storage phase of borophene corresponds to NaB_2_ with a theoretical specific capacity of 1240 mA h g^−1^, which is much larger than that of other two-dimensional materials. Most interestingly, sodium ion flows in the furrows of puckered borophene are extremely fast with a low energy barrier of 30 meV. Meanwhile, sodium diffusion on borophene was found to be highly anisotropic, as further verified by the results of the *ab initio* molecular dynamics simulations. The sodiated-borophene nanostructure shows enhanced electronic conductivity during the whole sodiation process, which is superior to other anode materials. Borophene is expected to be a promising candidate with high capacity and high rate capability for anode materials in sodium-ion batteries.

## Abstract 2

Two-dimensional boron synthesized by the chemical vapor deposition method is an atomically thin layer of boron with both light weight and metallicity.

## Graphical abstract

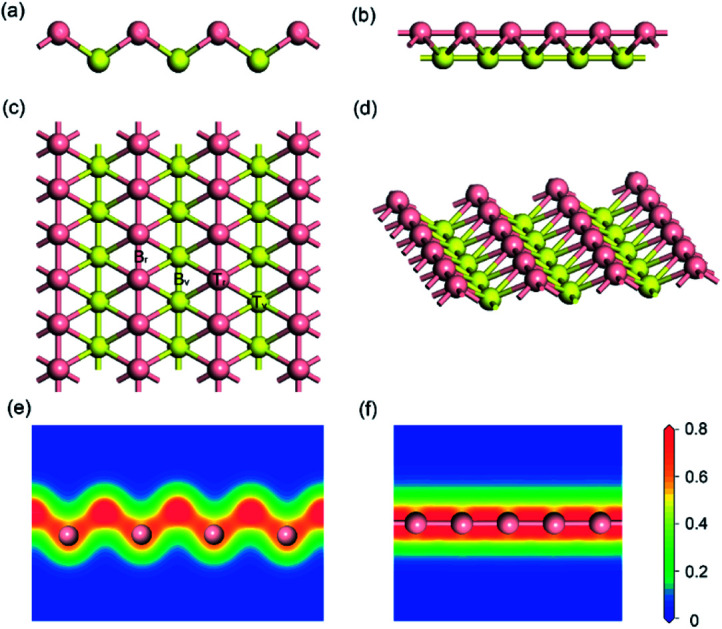

## 1. Introduction

Lithium-ion batteries (LIBs) have been widely applied in various applications for portable and telecommunication electronic devices due to their mature commercial technologies.^1–5^ However, faced with the increased demands of large-scale energy storage equipment, such as electric vehicles and power backups, concerns about the expensive cost of an Li-ion battery system are being reviewed. Very recently, intense scientific interest has been attracted to sodium-ion batteries (SIBs) because of their definite advantages of lower material costs and plentiful resources from the sea, compared to LIBs.^6–9^ Thus, in large-scale energy storage applications, SIBs are promising alternatives to replace LIBs. To date, in the pursuit of improvements in both storage capacity and rate capability, an extensive series of attempts have been launched to search for appropriate anodes for SIBs,^10–12^ and a few materials have been developed and successfully synthesized to be applied as anode electrodes. Among these, explorations for anode materials are not only limited to TiO_2_ oxides,^13–17^ alloys Sn–Sb^18–20^ or the traditional nongraphitic carbon-based materials, such as coke,^21^ carbon black,^22^ or template carbon,^23^ but have also turned to low-dimensional electrode materials, such as graphene and graphdiyne,^24–26^ carbon nanosheets,^27^ hollow carbon nanowires^28^ and nanospheres,^29^ which provide a high surface-volume ratio and unique electronic properties with large reversible capacity, high rate capability and long cycle life. Meanwhile, other novel low-dimensional materials are attracting increasing interest. For example, MoS_2_ nanoflowers were synthesized by Z. Hu *et al.*^30^ and used as a high performance anode in Na-ion batteries with high discharge capacities of 350 mA h g^−1^ at a current density of 0.05 A g^−1^, 300 mA h g^−1^ at 1 A g^−1^, and 195 mA h g^−1^ at 10 A g^−1^. Single-layer exfoliated MoS_2_ nanosheets exhibit a first discharge capacity of 376 mA h g^−1^, which is higher than that of pure MoS_2_ nanosheets and those (200–240 mA h g^−1^) of hard carbon materials.^31^

In late 2015, a new 2D material known as borophene (crystalline 2D boron sheets) was reported, which was successfully synthesized on an Ag (111) surface under ultrahigh-vacuum conditions by the groups of Oganov, Hersam, and Guisinger.^32^ More interestingly, borophene exhibits potential applications for an anode material in LIBs with an attractive high specific capacity of 1860 mA h g^−1^ and an extremely low energy barrier of 2.6 meV for Li diffusion due to its unique puckered morphology and metallic characteristics, which were confirmed by H. R. Jiang *et al.* with density functional theory (DFT) and *ab initio* molecular dynamics (AIMD) simulations.^33^ Inspired by the excellent performance of borophene as an anode material for LIBs, a question in our heads was whether it is possible to use borophene as the anode of SIBs. To the best of our knowledge, to date, hardly any research experimental effort on the sodium storage on borophene has been reported, even for theoretical calculations. To answer this question, a detailed study on the storage and diffusion of sodium on borophene is urgently needed.

To evaluate its potential as an anode material in SIBs and the effect of defects on the sodium storage and diffusion on borophene, herein, first-principles calculations based on density functional theory (DFT) and *ab initio* molecular dynamics (AIMD) simulations were firstly performed to examine the energetics and dynamics of sodium on both a perfect and a defective borophene monolayer. The structure of pure borophene was firstly optimized and the electron density data were also obtained. The adsorption energies and Mulliken charges of sodium on possible high-symmetry adsorption sites of both perfect and defective borophene monolayers were calculated. In order to locate the maximum theoretical sodium storage capacity, the most stable structures of sodiated borophene with increasing sodium concentration were investigated accordingly. By using both the LST/QST method and AIMD simulation, the migration process of sodium ions in two representative directions of the borophene monolayer was probed. Finally, the electronic conductivity of sodiated borophene was further investigated by calculating the electron density of states (DOS).

## 2. Computational methods

In this work, all calculations were performed using the Cambridge Sequential Total Energy Package (CASTEP) based on density functional theory (DFT). The Perdew–Burke–Ernzerhof (PBE)^34^ exchange function of the GGA was chosen for calculation and the ultrasoft pseudo potentials (USPP) introduced by Vanderbilt^35^ were employed for all the ion-electron interactions. Herein, B 2s^2^ 2p^1^ electrons and Na 2p^6^ 3s^1^ electrons were explicitly regarded as valence electrons. Convergence with respect to both energy cutoff and *k*-point mesh have been strictly tested. After a comprehensive consideration of the convergence tests and computational efficiency, an energy cutoff of 500 eV was chosen to ensure that the total energies converged within 1 × 10^−4^ eV per atom. Brillouin zone sampling for borophene was carried out with a 25 × 15 × 1 mesh using the Monkhorst–Pack method.^36^ For all the following supercell calculations, *k*-point meshes with the same density as the unit cell were used. The energy convergence criterion for the self-consistent field (SCF) calculation was set to 1 × 10^−6^ eV per atom.

In order to locate the most-stable configuration of borophene and sodium-stuffed borophene, atom and cell optimization were performed beforehand using total energy minimization methods. The total-energy difference was within 10^−6^ eV per atom, the maximum force was within 10^−4^ eV Å^−1^, the maximum stress was within 0.01 GPa and the maximum atom displacement was within 10^−4^ Å. Two-dimensional periodic boundary conditions were introduced to all the calculations for borophene and the sodiated borophene monolayer. Moreover, to simulate the monolayer and eliminate the interactions between adjacent conformations, a vacuum region of 20 Å was also applied in the direction perpendicular to the borophene and sodiated borophene plane.

The AIMD simulations were carried out with a 5 × 3 borophene supercell containing one sodium atom at a room temperature of 300 K using a statistical ensemble with a fixed particle number, volume and temperature (NVT). The time step was set to be 1 fs to ensure an energy drift of less than 1 meV per atom per ps. The system was first equilibrated for 8 ps and the following 2 ps were used for mean square displacement (MSD) analysis and trajectory sampling. An energy cutoff of 350 eV, a 3 × 3 × 1 *k*-point mesh and a periodic boundary condition were employed during the AIMD simulation.

## 3. Results and discussion

### 3.1 Structure of monolayer borophene

**Fig. 1:**
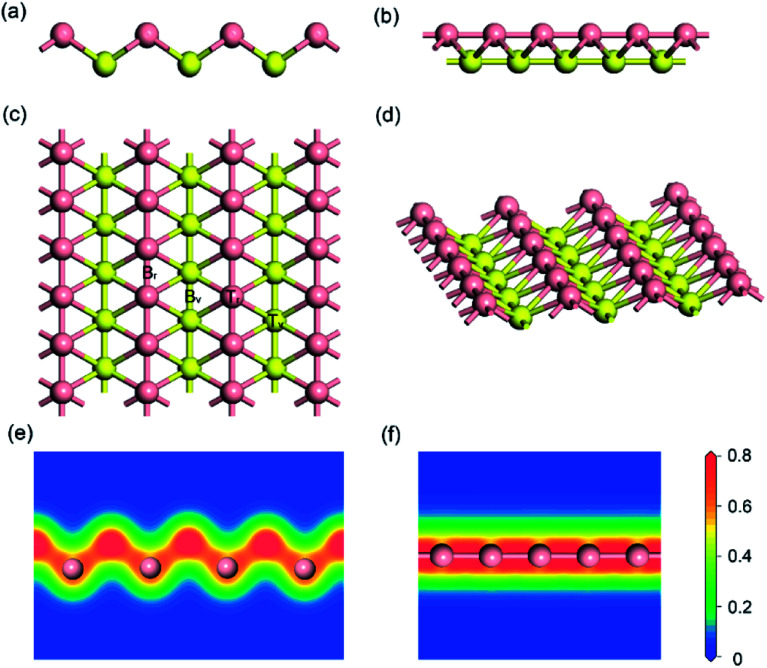
Side views (a) and (b); top view (c) and 3D view (d) of the optimized structure for (5 × 3) monolayer borophene; electron densities (unit: electrons per Å^3^) in (e) the [100] and (f) the [010] direction. For ease of visualization, the boron atoms in different atomic layers are marked in either pink or yellow.

Firstly, the structure of monolayer borophene was relaxed. Fig. 1 presents the structure of monolayer borophene after optimization and calculated electron densities. Unlike the 2D plate structure of graphene,^37,38^ borophene has a special puckered surface with two atomic boron layers (pink or yellow atomic layers in Fig. 1), and the corrugations along the *a*-direction are repeated. Our calculated lattice parameters of the unit cell are *a* = 1.610 Å and *b* = 2.858 Å, in fairly good agreement with the reported experimental and theoretical results,^32,33,39^ generally within 0.005 Å. In borophene, each boron atom is covalently bonded with six neighbors and resides in the center of a boron hexatomic-ring (top view of Fig. 1c), with two of them in the same atomic layer and the other four in the neighboring atomic layer, leading to a rhombohedral repeating unit. The optimized B–B bond length is 1.610 Å for those atoms in the same atomic layer and 1.873 Å for those atoms within different atomic layers. Accordingly, there are two kinds of B–B–B bond angles: one is 64.56° and the other is 50.88°. Moreover, viewed from the plot of electron densities (Fig. 1e and f), many electrons locate in the area between two boron atoms, indicating that all B–B bonds are covalent.

### 3.2 Defects in borophene

**Fig. 2:**
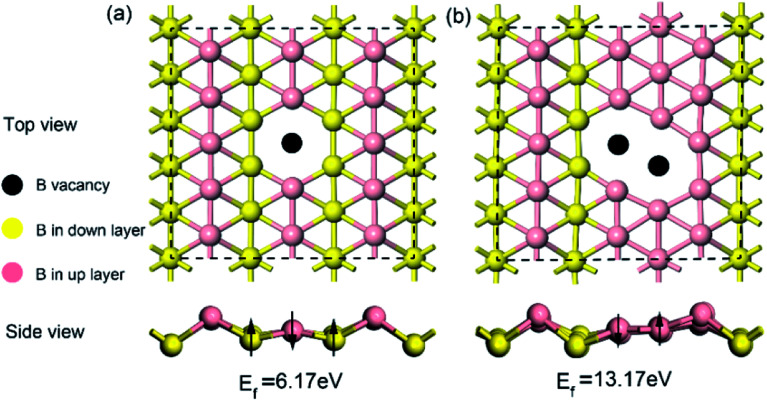
Top and side views of the optimized structures for (a) borophene with a single vacancy defect and (b) borophene with a double vacancy defect.

Given that intrinsic defects play a significant role in lithium storage and diffusion on 2D materials^40,41^ and the fact that defects may inevitably be produced in the process of synthesis, in this work, it is highly necessary to evaluate the effect of an intrinsic defect on sodium storage and diffusion on borophene. Because of the equivalency of all the boron atoms, there is only one type of single or double vacancy configuration before structural optimization. Therefore, removing one atom or two adjacent boron atoms from the (5 × 3) supercell creates a single or double vacancy defective configuration, respectively, for borophene without absorbed sodium. The optimized structures of monolayer borophene with single or double vacancy defects are depicted in Fig. 2a and b, respectively. Viewed from Fig. 2a and b, missing a B atom creates a six-membered ring with a hollow and the double vacancy defect forms an eight-membered ring with a bigger hollow, which may benefit sodium migration across borophene layers. Besides, it is worth noting that after introducing vacancy defects the special puckered phenomenon around the hollow is mitigated, and the local borophene surface becomes relatively flat, which may have a pronounced effect on the interaction between sodium and defect-containing borophene. The formation energy of a single vacancy defect in monolayer borophene is 6.17 eV, which is smaller than the same defects found in graphene (7.38 eV) but higher than that in silicenene (3.01 eV), indicating that this defect is relatively easily formed in borophene. However, the formation energy of a double vacancy defect is 13.17 eV, more twice than that of a single vacancy defect. The single vacancy defect in monolayer borophene exhibits more favorable stability than a double vacancy. Thus, the probability of two adjacent boron atoms simultaneously moving away from their original location to form one double vacancy is extremely small under normal operating conditions. In the following section, the effect of a single vacancy defect on sodium storage and diffusion in borophene is mainly discussed.

**Fig. 3:**
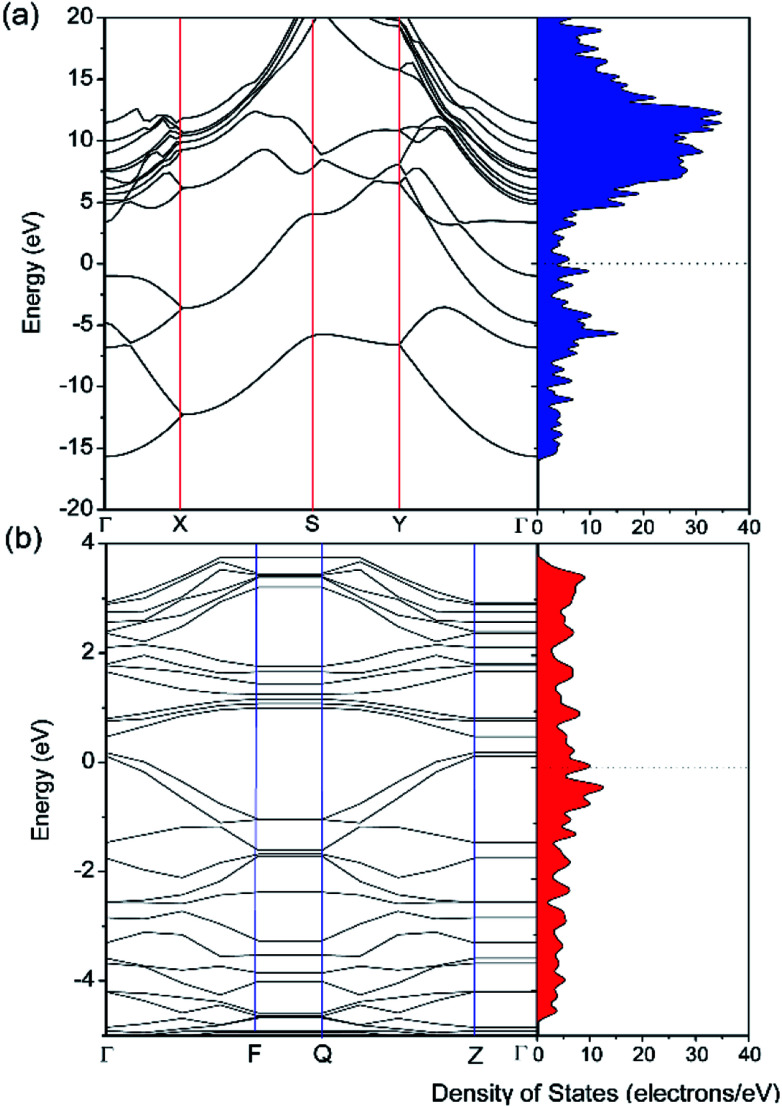
Calculated band structure and density of states (DOS) of (a) pristine borophene and (b) borophene with a single vacancy defect. The Fermi level is set at 0 eV.

The electronic properties of pristine and defective borophene were therefore studied by calculating their band structure and density of states (DOS), as depicted in Fig. 3. Unlike many other 2D materials exhibiting semi-conducting or semi-metallic characteristics,^40–44^ pristine and defective borophene are both intrinsically metallic due to having many electronic states across the Fermi level, which is also verified by previous work.^32^ Therefore, it is clear that pristine borophene shows excellent electronic conductivity as an electrode material even without any further structural modification or doping. Compared to pristine borophene, the electronic structure of defective borophene undergoes prominent changes as a consequence of a single vacancy defect. It is observed that after introducing vacancy defects, electronic states around the Fermi level significantly increase compared to those of pristine borophene due to a dangling bond introduced by a boron vacancy defect. Therefore, the metallic nature of defective borophene becomes more prominent.

### 3.3 Na adsorption on pristine and defective borophene

A fundamental requirement for a promising anode material for SIBs is that the material must have a relatively high specific capacity, especially in high power density application such as electric vehicles. It is known that the specific capacity is dependent mainly on large sodium storage. For borophene, the adsorption energy of sodium on the layer determines the specific capacity of the borophene material. To locate the most stable adsorption site of pristine borophene, the adsorption behavior of a single sodium atom on the four possible high-symmetry adsorption sites of (3 × 2) borophene was explored first: (a) T_V_ sites: over the top of boron atoms in the valley; (b) B_V_ sites: over the mid-point of B–B bonds in the valley; (c) T_R_ sites: over the top of boron atoms in the ridge; (d) B_R_ sites: over the mid-point of B–B bonds in the ridge, as shown in Fig. 1c. The stability of the sodiated borophene system can be estimated from their corresponding sodium average adsorption energy (*E*_a_). The adsorption energies were calculated with the following equation:^45,46^

1 *E*_a_ = [*E*_Na–borophene_ – (*E*_borophene_ + *E*_Na_)]

where *E*_Na–borophene_, *E*_a borophene_ and *E*_Na_ are the total energy of the sodiated borophene system, the borophene and a single sodium atom in a box, respectively.

**Fig. 4:**
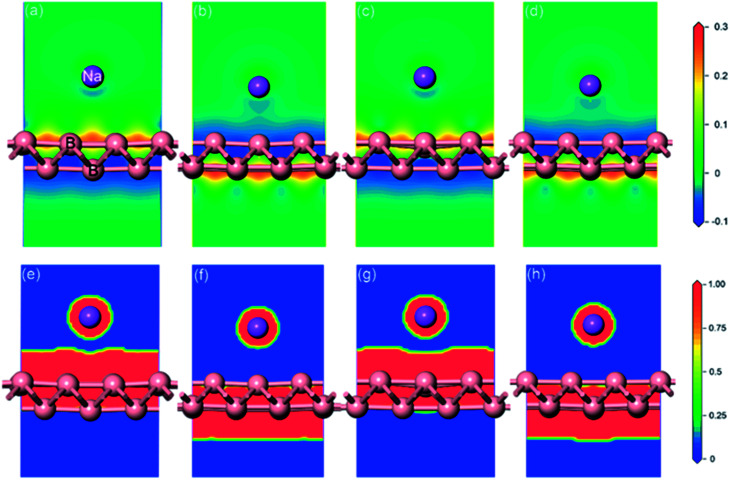
Charge density difference (a)–(d) and electron localization function (ELF) slice maps (e)–(h) of pristine monolayer borophene with four high-symmetry sites (B_R_, B_V_, T_R_, T_V_) absorbed with sodium. The red and blue areas represent electron gains and losses, respectively. The charge difference plot (in electrons per Å^3^) is calculated from *ρ* = *ρ*_total_ − *ρ*_borophene_ − *ρ*_Na_, where *ρ*_total_, *ρ*_borophene_ and *ρ*_Na_ are the total charge of the system, borophene and sodium atom, respectively. High ELF values show that at the examined position the electrons are more localized than in a uniform electron gas of the same density.

Full geometrical optimizations for the structure of sodium adsorption on pristine monolayer borophene were carried out. The calculated adsorption energies (*E*_ads_), adsorption height and Mulliken charge of a single sodium atom adsorbed on (3 × 2) borophene are listed in Table 1. It can be seen that the B_V_ site is the most stable one with an adsorption energy of −2.234 eV. In this case, the distance between the sodium atom and mid-point of the B–B bond in the valley is 2.91 Å. For the case of sodium adsorption on T_V_ sites, the corresponding adsorption energy is −2.233 eV. Interestingly, the difference between this value and that of the most stable B_V_ site is infinitesimally small and even negligible, indicating that the sodium adsorption on these sites along the valley is similar and may contribute to a low diffusion barrier along this direction. For a better understanding of this unusual phenomenon, the Mulliken charge analysis was calculated (in Table 1). The calculated results suggest that the electron transfer from sodium to pristine borophene at the B_V_ and T_V_ sites has comparable values, *i.e.*, 0.96 and 0.95|*e*|, respectively. Moreover, the adsorption energies of the sodium atom on the two ridge sites B_R_ and T_R_ are −1.949 eV and −1.957 eV, respectively, which are far smaller than those on the valley sites B_V_ and T_V_. To gain further insight into the nature of the sodium–borophene combination, the charge density difference maps of these four high-symmetry sites absorbed with sodium are depicted in Fig. 4a–d. It is found that electrons tend to locate around boron atoms and the electron density surrounding the sodium atoms is small, which agrees with the larger electronegativity of boron (2.04) than sodium (0.93). In addition, the extremely low values (about 0) of the electron localization function (ELF) in the areas between the sodium and boron atoms indicate the strong ionic nature of the Na–B bonds, as shown in Fig. 4e–h, consistent with the above analysis results from the Mulliken charge. It is a remarkable fact that lithium atoms adsorbing on the graphene surface would form lithium clusters, which limits the application of graphene as an anode material for LIBs.^47,48^ Thus, in this work, it is absolutely necessary to compare the adsorption energy of sodium atom on pristine borophene (−2.234 *vs.* sodium atom) with the cohesive energy of sodium bulk (−1.288 eV). The result reveals that sodium atoms are free of clusters and would form a stable 2D layer on the pristine borophene surface at low sodium concentration, which is beneficial to the safe operation in the process of charging and discharging of SIBs.

**Table 1:** Adsorption energies (*E*_ads_ in eV), adsorption height (in Å) and Mulliken charge of Na atoms (in *e*) of single sodium atom adsorbed on (3 × 2) pristine borophene

	*E*_ads_	Adsorption height	Mulliken charge
B_R_	−1.949	2.34	0.83
B_V_	−2.234	2.91	0.96
T_R_	−1.957	2.45	0.84
T_V_	−2.233	3.06	0.95

**Fig. 5:**
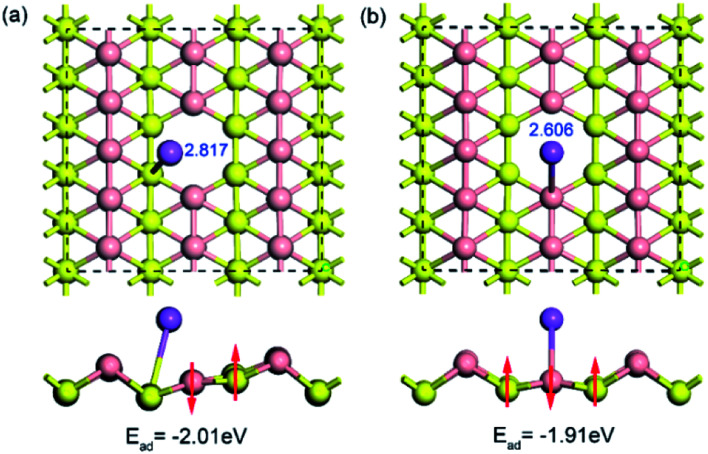
Top and side views of the optimized structures for a sodium atom adsorption on a 5 × 3 borophene supercell with a single vacancy defect.

To examine the influence of vacancy defects on sodium storage in borophene, the behavior of sodium adsorption on the sites around a single vacancy of borophene was investigated. Considering the high symmetry of borophene, two adsorption sites of T_V_ and T_R_ around the single vacancy were calculated, as depicted in Fig. 5a and b, respectively. The results show that the adsorption energy of sodium adsorption on the T_R_ site around the single vacancy is higher than that in the case of the T_V_ site, which is consistent with the cases of sodium adsorption on the pristine borophene surface. Moreover, the adsorption energies (−2.018 and −1.913 eV) of sodium adsorption on the defective borophene surface are higher than that of sodium adsorption on the pristine borophene surface with adsorption energies of −2.234 and −1.957 eV, indicating that defects in borophene can weaken sodium adsorption on it, so it is important to control the generation of defects in the synthetic production of borophene material.

### 3.4 Theoretical capacity of sodium storage on borophene

**Fig. 6:**
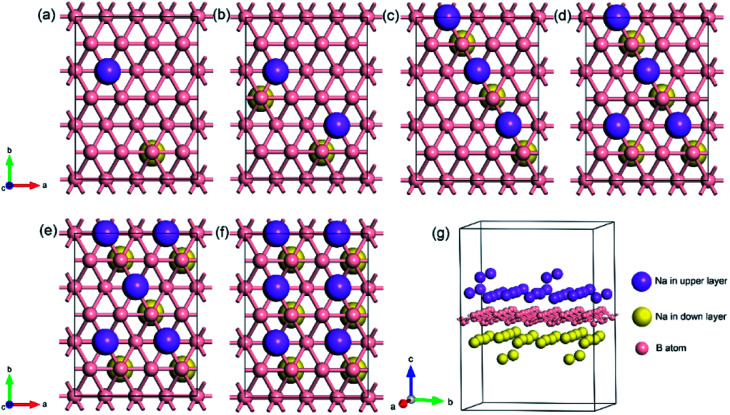
The most stable structures at different *x* values of Na_*x*_B_24_; the *x* value is an even number ranging from 2 to 14 for (a)–(g), respectively.

**Fig. 7:**
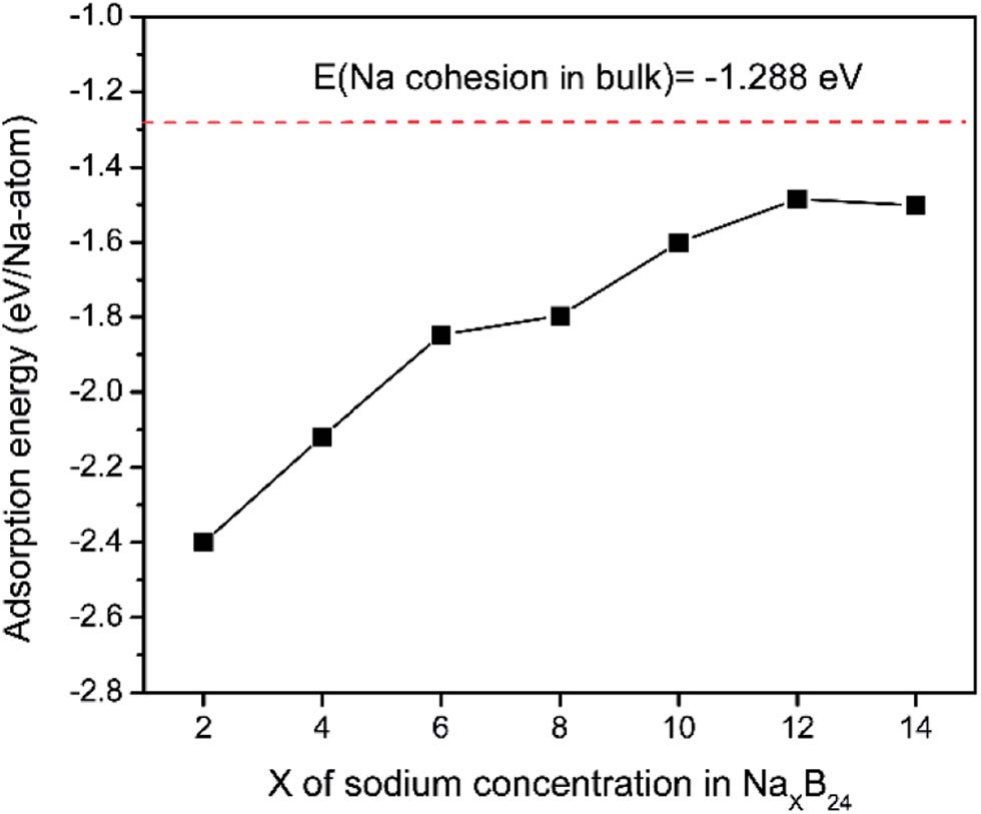
Adsorption energies as a function of sodium concentration *x* in Na_*x*_B_24_; the *x* value is an even number ranging from 2 to 14.

To explore the theoretical maximum capacity of sodium storage on borophene, the sodium adsorptions on borophene at different concentrations were investigated by gradually loading sodium atoms on them. The 4 × 3 supercells of borophene were used for the following adsorption calculations. Here, the *x* values of Na_*x*_B_24_ are even numbers ranging from 2 to 14. For each concentration, several possible configurations were explored. The most stable structure at each *x* value of sodium concentration are depicted in Fig. 6a–g and the corresponding average adsorption energies are shown in Fig. 7. It is found that the sodium atom prefers to locate on the T_V_ site when *x* = 2, and the average adsorption energy (−2.4 eV) of two sodium atoms residing on the same side of borophene is higher than −2.12 eV for the case of two sodium atoms residing on the upper and lower surfaces (Fig. 6a), which means the two sodium atoms adsorb preferentially on the two sides of borophene to reduce the electrostatic repulsion between them. With an increase in sodium concentration, the adsorption energy gradually reduces (Fig. 7), which indicates a reduction in thermodynamic stability during the process of continuous sodium adsorption. The decrease in average adsorption energy may be due to the fact that the weak electrostatic attractions between the borophene host and the sodium adatoms are gradually beaten by the enhanced Na–Na electrostatic repulsion at relatively high sodium concentration.

The fully sodium storage phase of borophene should meet the following four conditions: (a) it should have maximum sodium concentration; (b) the average adsorption energy for the sodium atom should be less than zero; (c) there should be no sodium atom extrusion out of the borophene surface; (d) there should be no irreversible deformation of the structure of borophene.^40,49^ Interestingly, sodium atoms can reside almost anywhere in the furrows of borophene with a negligible adsorption energy difference (<0.01 eV). Thus, the maximum sodium concentration is dependent only on the minimum distance between sodium atoms in the furrows. By examining all the optimized structures and corresponding adsorption energies, the maximum concentration of sodium adsorption on a 4 × 3 supercell is *x* = 12, corresponding to Na_12_B_24_, as depicted in Fig. 6f. The adsorption energy of the Na_12_B_24_ system is −1.485 eV/Na atom, lower than the cohesive energy of Na bulk of −1.288 eV/Na atom, which means that sodium atoms are free of clusters which is beneficial to safe operation in the process of sodiation. In the configuration of Na_12_B_24_, both upper and lower surfaces of borophene are completely covered with sodium atoms, which all reside in the furrows. Although the adsorption energy for the case of *x* = 14 is negative and lower than the cohesive energy of Na bulk, with the addition of more sodium atoms into the furrows, the added sodium atoms are decidedly extruded out of the furrow of borophene, forming a second sodium layer on the surface of the first absorbed sodium layer, as evidenced in Fig. 6g. Moreover, the stability and reversible deformation of borophene during the repeated sodiation and disodiation were investigated using the method proposed by Tritsaris *et al.*, in which all the sodium atoms were removed and the structure was again optimized.^50^ Calculation shows that after removing all sodium atoms, the structure of borophene is not destroyed and can be restored to its initial structure. All the calculated results for Na_12_B_24_ meet the above four conditions for a fully sodium storage phase; therefore, the maximum sodium concentration using a 4 × 3 borophene supercell corresponds to NaB_2_ with a theoretical specific capacity of 1240 mA h g^−1^.

**Fig. 8:**
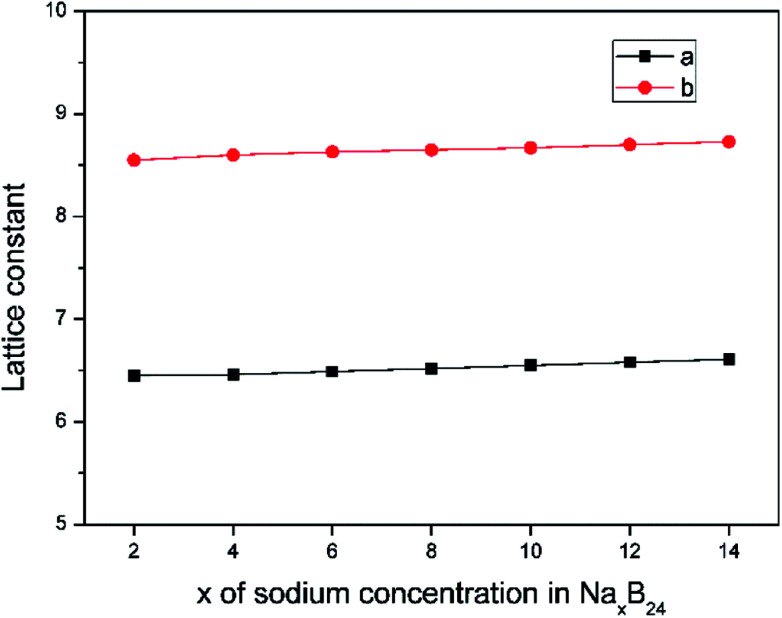
Lattice constant as a function of sodium concentration *x* in Na_*x*_B_24_, where the *x* value is an even number ranging from 2 to 14.

The lattice parameters of borophene at different sodium concentrations are shown in Fig. 8. Fig. 8 indicates that even at a concentration as high as Na_0.5_B, the volume expansion is less than 2%. The fluctuations of lattice parameters along with the sodiation process are small, indicating good structural stability when used as an anode material for sodium-based batteries.

### 3.5 Na diffusion on borophene

**Fig. 9:**
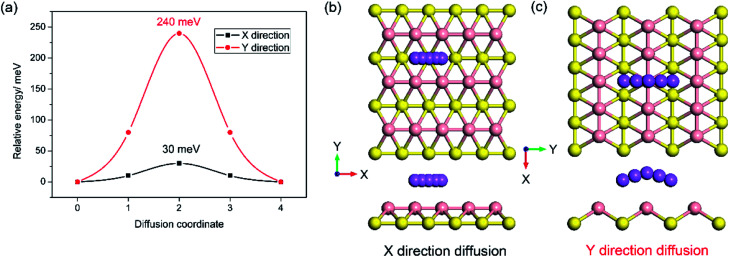
Energy profile of sodium diffusion in the *X* direction in the furrows and the *Y* direction crossing ridges (a); top and side views of sodium diffusion in the furrows (b) and crossing the ridges (c).

In addition, to the high specific capacity, a good rate capability is another requirement for a promising anode material for SIBs, especially in high power density applications such as electric vehicles. It is known that the rate capability is dependent mainly on the transport properties of sodium ions and electrons, corresponding to the sodium diffusion barrier and electronic conductivity of sodiated-borophene. In this work, the linear synchronous transit/quadratic synchronous transit (LST/QST) method combined with the conjugate gradient (CG) refinements was adopted for the calculation of energy barriers of the transition states (TS) and intermediates for Na ion migration.^51^ Considering the high symmetry of borophene, two main diffusion pathways were calculated: one is the path in the furrows along the *X* direction, and the other is the path crossing the ridges which is perpendicular to the furrows, along the *Y* direction, as depicted in Fig. 9b and c, respectively. The corresponding energy profiles of sodium diffusion along these two pathways are shown in Fig. 9a. The results show that sodium migration along the *Y* direction has an activation energy barrier of 240 meV, corresponding to the transition state located on the top of the T_V_ adsorption site between two adjacent B_V_ sites. In contrast, a diffusion energy barrier of only 30 meV is found for a sodium hopping between two B_V_ sites along the furrows in the *X* direction, much lower than that along the diffusion path in the *Y* direction. This ultra-low diffusion barrier is due to the fact that all adsorption sites in the furrows have an extremely similar sodium adsorption state, indicating that the sodium atoms find it extremely easy to diffuse along the furrows. To verify the accuracy of our calculations of the energy barrier using the LST/QST method, other diffusion paths starting from the TS and moving down toward the minima along both the reactant and the product directions were also calculated. As expected, no energy minima were found on those paths other than the reactant and product. It is worth noting that our calculations of the sodium diffusion barrier are based on the results at 0 K, but thermal vibrations in the sodiated borophene system cannot be ignored under the actual working conditions of NIBs. To evaluate the effect of temperature on the molecular transition rate, the diffusion coefficient was calculated from one-sixth of the slope of the curve of MSD from AIMD.

**Fig. 10:**
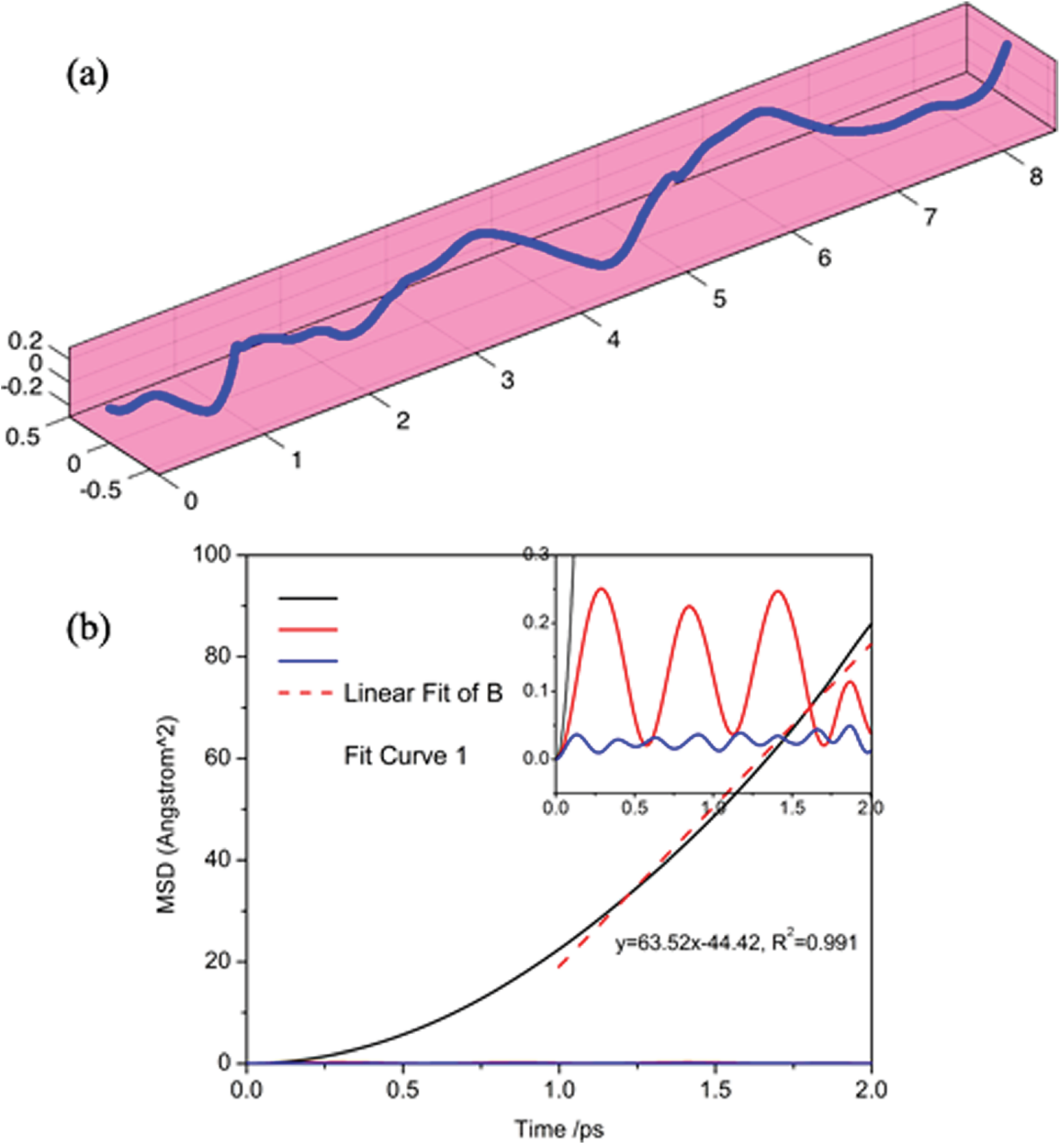
Calculated trajectories (a) and MSD in three directions (b) of a sodium ion during the last 2 ps by AIMD simulation.

To further verify the ultra-fast, highly anisotropic sodium migration on borophene, AIMD simulations at 300 K were also carried out subsequently, which are flexible for studying any system and provide accurate dynamic models.^52^ The calculated MSD in three directions of the sodium ion during the last 2 ps of the simulation is depicted in Fig. 10b. It is found that the *Y*- and *Z*-coordinates of the sodium ion are relatively restricted within a small range, indicating that diffusion along a direction perpendicular to the furrow of the sodium ion is prohibited by the relatively higher diffusion barrier. In contrast, the sodium ion is allowed to move almost freely along the furrow direction (*X*-direction) with an extremely large diffusion coefficient of 6.35 × 10^−3^ cm^2^ s^−1^ at 300 K. which is verified by the direct observation of sodium diffusion trajectories presented in Fig. 10a. Viewed from the last 2 ps of the sodium ion trajectory, the sodium ion is allowed to drift through the entire furrow, while there is no visualization of a sodium ion jumping across the ridges to neighboring sites. Interestingly, unlike most other diffusion processes consisting of long lattice vibrations followed by abrupt jumps,^33^ sodium ion flow in the furrows of borophene is nearly ballistic with a nearly negligible migration barrier.

### 3.6 Electronic structure of a sodiated-borophene nanostructure

**Fig. 11:**
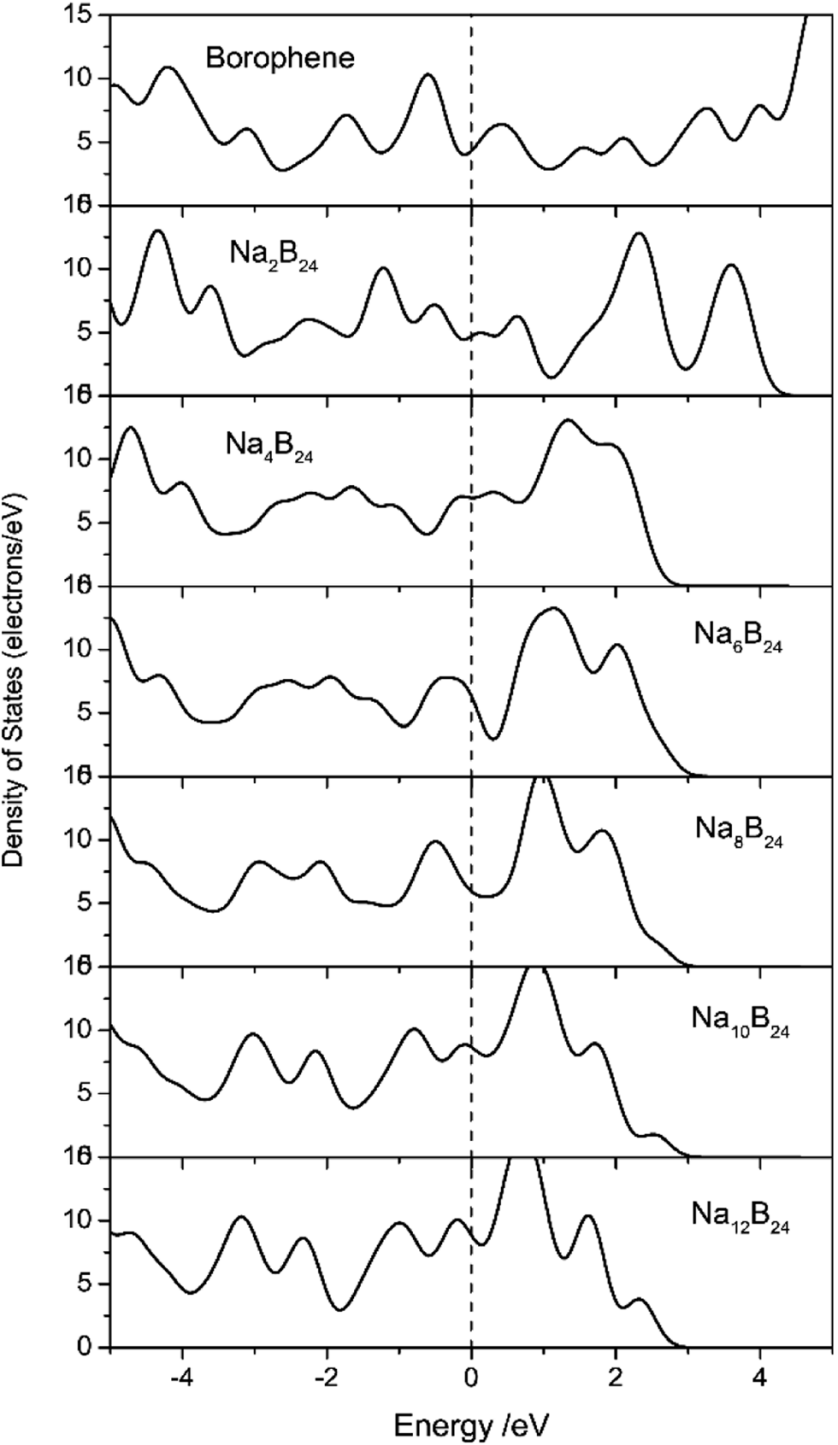
Density of states (DOS) of pristine borophene and sodiated-borophene system. The Fermi levels were shifted to zero.

Electronic transport is another important factor that determines the rate capability of anode materials in SIBs; therefore, the electronic structure of the sodiated-borophene nanostructure was investigated by performing calculations of the density of states (DOS), as presented in Fig. 11. Unlike many other famous 2D materials that show a semi-conducting nature,^40,41,43,44,53,54^ pristine borophene is intrinsically metallic with a large number of electronic states around the Fermi level, which is also verified by some published works.^32,33^ Thus, we can be confident that the electronic conductivity of pristine borophene is enough for it to be used as an anode material in SIBs even with no further structural modification. Moreover, the DOS of these structures during the sodiation and disodiation process were also calculated in case the introduced sodium atoms could influence the electronic structure of the pristine phase. Viewed from Fig. 11, during the sodiation process of increasing sodium concentration, the electronic structures undergo obvious changes as a consequence of electron transfer from sodium to the borophene substrate. Not only is the metallic characteristic of the sodiated borophene nanostructure maintained, but the electronic conductivity is also enhanced during the continual sodiation process.

### 3.7 Comparison with other anode materials

To make a more comprehensive evaluation of the borophene material, the properties of theoretical specific capacity, diffusion barrier and electronic conductivity of some other current widely investigated anode materials for sodium-ion batteries from the literature are summarized in Table 2. It can be seen that borophene has the third largest theoretical specific capacity, the lowest diffusion barrier and the best electronic conductivity among all these listed anode materials. Despite the theoretical specific capacity of borophene being twice as low as that of Na_3_P and the sodium ion diffusivity on borophene being slightly superior to it, the electronic conductivity is much faster than that of Na_3_P with a semiconducting characteristic, indicating the much better rate capability of borophene. Furthermore, the theoretical specific capacity of borophene is also lower than that of the SnO_2_ based material, but its electronic conductivity has a semiconducting characteristic. Compared to other 2D materials with semiconducting characteristics, the specific capacity of borophene is almost 2.5 times that of graphyne and almost 4 times that of phosphorene and MoS_2_. In addition, the sodium ion diffusion on borophene is estimated to be faster than that on graphyne, phosphorene or MoS_2_ at room temperature. All the evidence demonstrates that borophene is a promising candidate with high capacity and high rate capability for the anode material in sodium-ion batteries.

**Table 2:** Summary of theoretical specific capacity (mA h g^−1^) and diffusion barrier (meV) of some currently widely investigated and promising anode materials for sodium-ion batteries

Compound	Theoretical specific capacity	Diffusion barrier	Electronic conductivity	Reference
Borophene	1240	30	Metallic	This work
Graphyne	558	400	Semiconducting	26 and 54–56
Phosphorene	324	40–63	Semiconducting	40, 41 and 56
MoS_2_	335	110	Semiconducting	44 and 57
Na_3_Ti_2_(PO_4_)_3_	133	750	Semiconducting	58–60
NaSi	954	310	Semiconducting	61–63
NaGe	369	270	Semiconducting	61–63
Na_15_Sn_4_	847	260	Metallic	61 and 64–66
Na_3_Sb	660	210	Semiconducting	64, 66 and 67
Na_8_SnO_2_	1378		Semiconducting	68
Na_3_P	2596	40	Semiconducting	64 and 69–72

## 4. Conclusions

First-principles calculations based on DFT and AIMD simulations were performed to explore the potential of borophene as an anode material in SIBs. It is found that after introducing vacancy defects, the special puckered structure becomes relatively flat and the metallic nature of the defective borophene becomes more prominent, while defects in borophene may weaken sodium adsorption. Therefore, it is important to control the generation of defects in the synthetic production of borophene. A single sodium atom is preferentially absorbed on the B_V_ site with a large adsorption energy with a value of −2.234 eV. The adsorption energies per sodium atom gradually reduce with an increase in the sodium concentration due to the enhanced Na–Na electrostatic repulsion. Moreover, the fully sodium storage phase of borophene corresponds to NaB_2_ with a theoretical specific capacity of 1240 mA h g^−1^, which is much larger than that of other 2D materials. Interestingly, sodium ion flow in the furrows of puckered borophene is extremely fast with a low energy barrier of 30 meV, which is much lower than those of other widely investigated anode materials such as graphyne (400 meV), phosphorene (40–63 meV), Na_3_P (40 meV), NaSi (310 meV) or MoS_2_ (110 meV). Meanwhile, sodium diffusion on borophene was found to be strongly anisotropic, as further verified by the results of AIMD that showed that sodium atoms can move freely in the furrows and rarely jump to the neighboring furrows. The sodiated-borophene nanostructure shows enhanced metallic characteristics and excellent electronic conductivity during the whole sodiation process, which is superior to other widely investigated anode materials with semiconducting characteristics. Considering these excellent performances, it is expected that borophene will be a promising candidate with high capacity and high rate capability for the anode material in sodium-ion batteries. More speculatively, borophene could also be an outstanding anode material for other metal-ions (*e.g.*, K-ion, Mg-ion, Ca-ion and Al-ion) batteries and the computational method used here is transplantable.

## Conflicts of interest

There are no conflicts to declare.

## Supplementary Material
